# Hitching a ride: Bamboo mosaic virus satellite RNA hijacks the methyltransferase fibrillarin for a ride across the plant

**DOI:** 10.1093/plcell/koaf226

**Published:** 2025-09-24

**Authors:** Yu-Hung Hung

**Affiliations:** Assistant Features Editor, The Plant Cell, American Society of Plant Biologists; Spearhead Bio, St.Louis, MO 63132, USA

Imagine you are stranded on the side of a rural road; you do not own a car, cannot call a taxi, and have no bus in sight. Your only chance of getting anywhere is to hitch a ride with someone passing by. This is much like how some viruses or subviral agents move around inside a plant.

A plant is made of millions of cells, each surrounded by a cell wall. Yet, these cells are not isolated; they are connected by microscopic channels called plasmodesmata (PD) (Kragler 2013). If you think of plant cells as many houses, the PDs are like sidewalks between plant cells, allowing nutrients, molecules, and occasionally viruses to pass through. To travel beyond the local neighborhood, plants have a long-distance transport system known as the vascular system, which moves sugars, hormones, and other materials from tissue to tissue. This system is like a subway system in a plant. And once inside, viruses can ride it to far-reaching destinations such as roots, flowers, and developing seeds. This is where real damage can occur, disrupting photosynthesis, growth, or reproduction (Harries and Ding 2011).

Despite their minimalistic nature, viruses can spread efficiently in a plant. They hijack the host cellular machinery both to replicate and to move around. Satellite RNAs take parasitism a step further: as subviral agents, they are entirely dependent on a helper virus for their replication, encapsidation, and efficient movement within the plant (Hu et al. 2009). However, the satellite RNA of Bamboo mosaic virus (satBaMV) has proven to be a particularly intriguing exception, demonstrating an impressive ability to move systemically through the plant even in the absence of its helper virus (BaMV) (Chang et al. 2016). This remarkable self-sufficiency, mediated by its encoded nonstructural 20-kDa protein (P20), hints at a deeper interaction with the host transport system. A new study by **Chih-Hao Chang and colleagues (Chang et al. 2025)** unravels the intricate molecular mechanism behind this phenomenon, revealing how satBaMV “hitches a ride” by exploiting a fundamental host process: fibrillarin (FIB)-mediated protein methylation.

Previous work by Chang et al. (2016) established that satBaMV's autonomous long-distance trafficking is dependent on its P20 protein, which forms a ribonucleoprotein (RNP) complex with satBaMV RNA. Importantly, this movement also requires the host nucleolar protein FIB, a highly conserved protein primarily known for its role in RNA processing and nuclear architecture (Rodriguez-Corona et al. 2015). FIB was previously found to co-precipitate with P20 and to be essential for satBaMV's long-distance movement (Chang et al. 2016). P20 itself was observed to localize to the nucleolus (where FIB resides) (Vijayapalani et al. 2009) and to form punctate structures at the PD. Now, Chang et al. (2025) look into the molecular details of the P20-FIB interaction and uncover a critical post-translational modification. Chang et al. demonstrate that FIB is not merely a binding partner but acts as a methyltransferase (MTase) that directly methylates P20 both in vitro and in vivo. The integrity of FIB's MTase catalytic triad is indispensable for this methylation (Chang et al. 2025).

The implications of this methylation are profound for satBaMV's journey: First, the motif where P20 gets methylated is critical for guiding P20 to the nucleolus, where FIB-mediated methylation happens. Nuclear localization of P20 is crucial for its methylation and, consequently, for the long-distance transport of satBaMV. Second, P20 methylation significantly enhances its ability to target the PD. Mutants of P20 that cannot be methylated show a significant reduction in PD localization compared with wild-type or methylation-mimic variants. Third, the methylation of P20 triggers a fascinating phenomenon: nucleocytoplasmic shuttling of FIB itself, along with P20, as part of the mobile RNP complex, directing it toward the PD. Finally, the MTase activity of FIB is essential for satBaMV's long-distance transport. Nonmethylated P20 mutants fail to facilitate systemic satBaMV trafficking, despite maintaining in vivo RNA binding affinity. This highlights that the crucial role of P20 methylation lies not in RNA binding per se but in coordinating the trafficking machinery.

In summary, satBaMV has evolved a clever strategy: it encodes a protein (P20) that, once methylated by the host's FIB within the nucleus, acts as a molecular “subway ticket.” This methylation enables the P20-FIB-satBaMV RNP complex to navigate efficiently from the nucleus to the cytoplasm, then to the PD, and then eventually into the vascular system, facilitating its plant-wide spreading (Figure). This discovery provides unprecedented mechanistic insight into how subviral agents leverage host protein modifications to overcome cellular barriers and achieve systemic infection.

**Figure.**
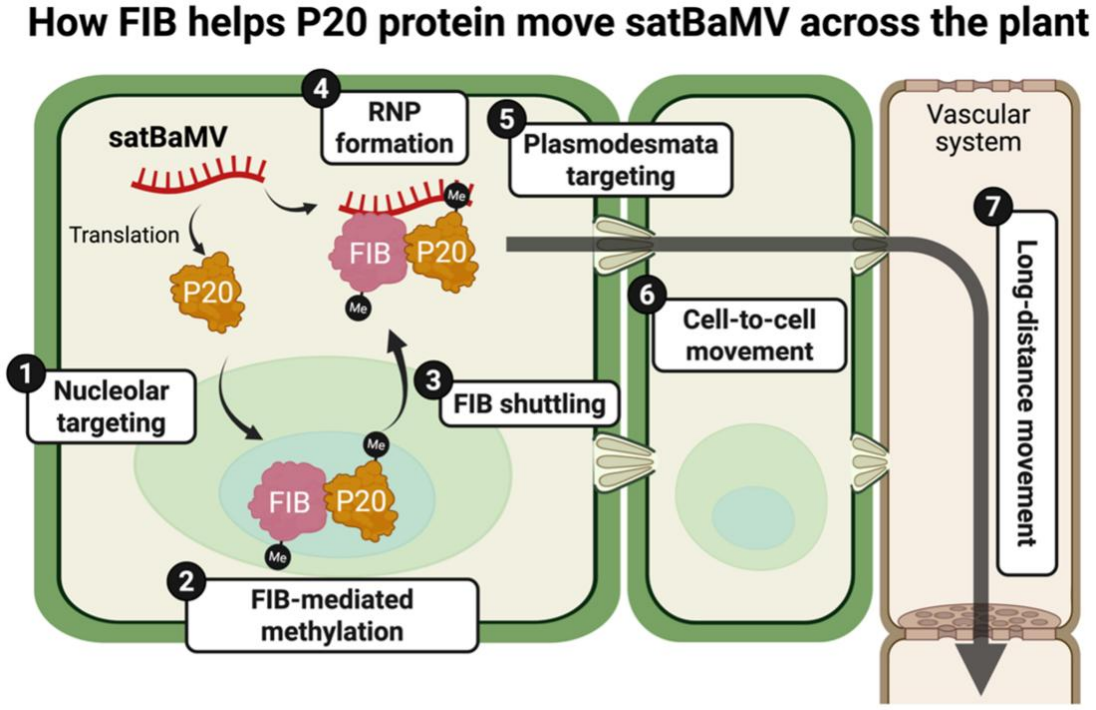
Hitching a ride: SatBaMV hijacks the host methyltransferase FIB to facilitate its movement from nucleus to cytoplasm, PD, and then eventually into vascular system. Modified from Chang et al. (2025), Figure 8 using BioRender.

## Recent related articles in *The Plant Cell*

- (Ma et al. 2022) revealed that some RNAs, including viroids, can move from the cytoplasm back into the nucleus, challenging the traditional 1-way view of RNA trafficking. In plants, Importin alpha-4 (IMPa-4) and VIRP1 mediate this nuclear import through recognition of a conserved C-loop RNA motif, which is also present in certain satellite RNAs.
- (Taliansky et al. 2023) discuss how plant Cajal bodies (CBs), as highly complex and multifunctional biomolecular condensates, are involved in a surprisingly diverse range of molecular mechanisms that are just beginning to be appreciated.

## Data Availability

No new data were generated or analysed in support of this research.
